# Therapeutic Notes

**Published:** 1905-01

**Authors:** Nelson W. Wilson

**Affiliations:** Buffalo, N.Y.


### PROGRESS IN MEDICAL SCIENCE.

### Therapeutic Notes.

CONDUCTED BY NELSON W. WILSON, M. D., Buffalo, N. Y.

FOR UREMIC CONVULSIONS.

℞ Tinct. veratri viridis .............................. 30.00 [℥i.]

Sig.—Fifteen to twenty drops hypodermatically and repeat half-hourly a dose of ten or twelve drops as conditions indicate.—*Norwood.*

The hypodermic use of this drug certainly cannot be seriously objected to, for in this most alarming and distressing of conditions, one will do most anything. At the same time, if one uses veratrum, the intelligent use of croton oil, mixed with butter and placed on the tongue, to melt and be swallowed reflexly if the patient is unconscious, will do wonders in aiding the elimination of the poisons which are doing the damage.

---

### IRRITABILITY OF THE BLADDER AFTER CONFINEMENT.

℞ Salol
Tinct. hyoscyamus, of each .......................8.00 [ʒii.]
Infusion buchu, to make ......................180.00 [℥vi ]

Mix. Dose—Teaspoonful three times a day.—*Fothergill.*

---

### FOR MALARIA.

THE *Medical Review of Reviews* gives the following much credit for its efficiency in malaria:

℞ Quinine sulphate...........................5.33 [gr. lxxx.]
Reduced iron..............................2.66 [gr. xl.]
Ext. nux vomica ........................... .66 [gr. x.]
Ext. belladonna............................ .26 [gr. iv.]

Mix and make into 40 pills. Dose—One pill three times a day (after meals).

A prescription which gave most excellent results in the treatment of malaria in soldiers returning from Cuba after the Spanish war, is as follows:

℞ Salicylate of cinchonidia...................... 12.00 [ʒiii.]
Essence of pepsin............................120.00 [℥iv.]

Mix. Dose—Teaspoonful four times a day. "Shake" label.

In connection with this aloin, strychnine and belladonna tablets are given each night to regulate the bowel action. Under the cinchonidia, the plasmodia disappeared rapidly in acute cases, and even in long standing cases where large quantities of quinine had been used, a cure was effected. After the disappearance of the plasmodia from the blood, as determined by microscopy, a course of tonic treatment by the administration of nux vomica, compound tincture of cinchona and compound tincture of gentian usually completed the cure.

---

### GLYCO-THYMOLINE IN NASAL CATARRH.—THE KRESS & OWEN BERMINGHAM NASAL DOUCHE.

ONE of the objects in the application of glyco-thymoline to the nasal cavity is to retain it in direct contact with the membrane for at least two minutes. This can be done very simply and effectively as follows: put into the douche one or two teaspoonfuls of glyco-thymoline, filling it with warm water, never using cold; then, with the index finger over the inlet control the flow, insert the nozzle into the nostril and hold the head well back. While allowing the solution to run into the nose, breathe through the mouth; this closes up the passage into the throat and enables the person to fill the entire nasal cavity. As soon as it is full, take the douche away, pinch the nostrils together and throw the head well forward; hold the solution in the nasal cavity for a minute or two and repeat in the other nostril. Clear the head gently to avoid forcing products of inflammation into the Eustachian tubes, as the glyco-thymoline loosens all the catarrhal crusts.

Do not blow the nose until you have thoroughly cleared the nose and throat. If the catarrhal condition affects the throat, gargle with one or two teaspoonfuls of glyco-thymoline diluted with a tablespoonful or two of hot water.

